# Becker Implant Intracapsular Rupture with Contralateral Axillary Silicone Lymphadenopathy in an Asymptomatic Patient: A Case Report and Literature Review

**DOI:** 10.7759/cureus.7638

**Published:** 2020-04-11

**Authors:** Scott A Kreitzberg, Daniel Sherbert, Jeffrey DeSano

**Affiliations:** 1 Plastic Surgery, Beaumont Health, Farmington HIlls, USA; 2 Plastic Surgery, Beaumont Health, Royal Oak, USA

**Keywords:** silicone lymphadenopathy, implant rupture, becker implant, contralateral axillary silicone lymphadenopathy

## Abstract

Silicone gel implants are widely used for cosmetic and reconstructive breast surgery. There has been a paradigm shift with increased utilization of implant-based breast reconstruction compared to autologous reconstruction in the United States over the past couple of decades. Implant rupture is a known complication of silicone gel implants with variable incidence and increased propensity with the age of the implant. Usually, the clinical findings suggestive of implant rupture are not obvious to the patient and surgeon. Intracapsular implant rupture, when the shell of the implant ruptures but the fibrous capsule formed by the breast remains intact, occurs in the majority of cases. While extracapsular rupture, which denotes silicone leakage extending beyond the capsule, is less common. In rare cases, silicone migrates beyond the capsule to distant sites, regional sites, and lymph nodes, leading to a variety of symptoms. Following mastectomy with lymph node dissection, the disruption of normal breast lymphatic drainage may result in aberrant drainage to internal mammary nodes and contralateral axillary lymph nodes. We present a unique case of axillary silicone lymphadenopathy due to contralateral breast intracapsular implant rupture in a patient with no previous ipsilateral breast surgery. The condition was found during a routine breast cancer screening. We also engage in a review of the relevant literature.

## Introduction

There has been a gradual increase in immediate and delayed breast reconstruction following mastectomy in the United States over the past few decades. This has been attributed to several factors, including the implementation of the Women’s Health and Cancer Rights Act (WHCRA) in 1998, which added mastectomy-related reconstruction services to payer benefits [1]. Implant-based breast reconstruction, as of 2002, has become the most common method of reconstruction in the United States [2]. According to the Nationwide Inpatient Sample (NIS) Database for patients with invasive breast cancer treated with mastectomy and immediate breast reconstruction between 2005 and 2012, roughly 78% opted for implant-based reconstruction [3]. Patient satisfaction with implant-based breast reconstruction remains high, despite local complications, possible need for reoperation, and potential for implant rupture [4].

Implant rupture and the potential harm from free silicone have been under scrutiny for years. The incidence of implant rupture increases with implant age, and the actual prevalence in asymptomatic patients is unknown [5]. According to data from Collis et al., based on examining third-generation implants utilizing MRI for rupture identification, ruptures usually begin six to seven years after placement. Also, the rupture rate was reported to be 11.8% at 13 years [6]. This is consistent with the findings of Holmich et al. who identified a 2% rupture rate at five years and 15-17% at 10 years [7]. Clinically, most silicone implant ruptures are not obvious but rather silent and detectable only by imaging modalities. The sensitivity of plastic surgeons to diagnose rupture is estimated to be around 30% [8]. Breast symptoms and abnormal physical examination may lead to the identification of an implant rupture; however, a more accurate diagnosis is accomplished with imaging techniques including ultrasonography, mammography, CT, and MRI. MRI is considered the imaging modality of choice for silicone gel implant rupture in post-mastectomy patients [9]. Extracapsular leakage can lead to silicone granulomatous formation and, on rare occasions, migration to distant sites and regional lymph nodes [10]. Based on the literature review, silicone lymphadenopathy has only been identified in fewer than 180 cases [11,12]. Given the increase in implant-based breast reconstruction for breast cancer in the United States over the past two decades and the increased incidence of implant rupture with age, it is important to recognize silicone migration as part of a differential diagnosis for ipsilateral as well as contralateral lymphadenopathy discovered clinically or radiographically.

## Case presentation

An asymptomatic 71-year-old female with a history of recurrent right breast ductal carcinoma in situ status post-mastectomy and radiation with right implant-based reconstruction 15 years prior and no left breast surgery presented for routine screening mammography. The findings revealed enlarged axillary lymph nodes. Her past surgical history was significant for right breast partial mastectomy and radiation therapy in 1990, at age 44, for ductal carcinoma in situ. In 2001, she had recurrent right breast ductal carcinoma in situ and underwent a right mastectomy, axillary lymph node dissection, and immediate reconstruction with an associated submuscular tissue expander placement. Following routine expansion, in 2002, the tissue expander was removed with capsulectomy, and a dual lumen breast implant was placed (Mentor Siltex Round Becker 50 cohesive I; Mentor Worldwide LLC, Irvine, CA), filled with 275 cc of saline and 200 cc of silicone.

**Figure 1:**
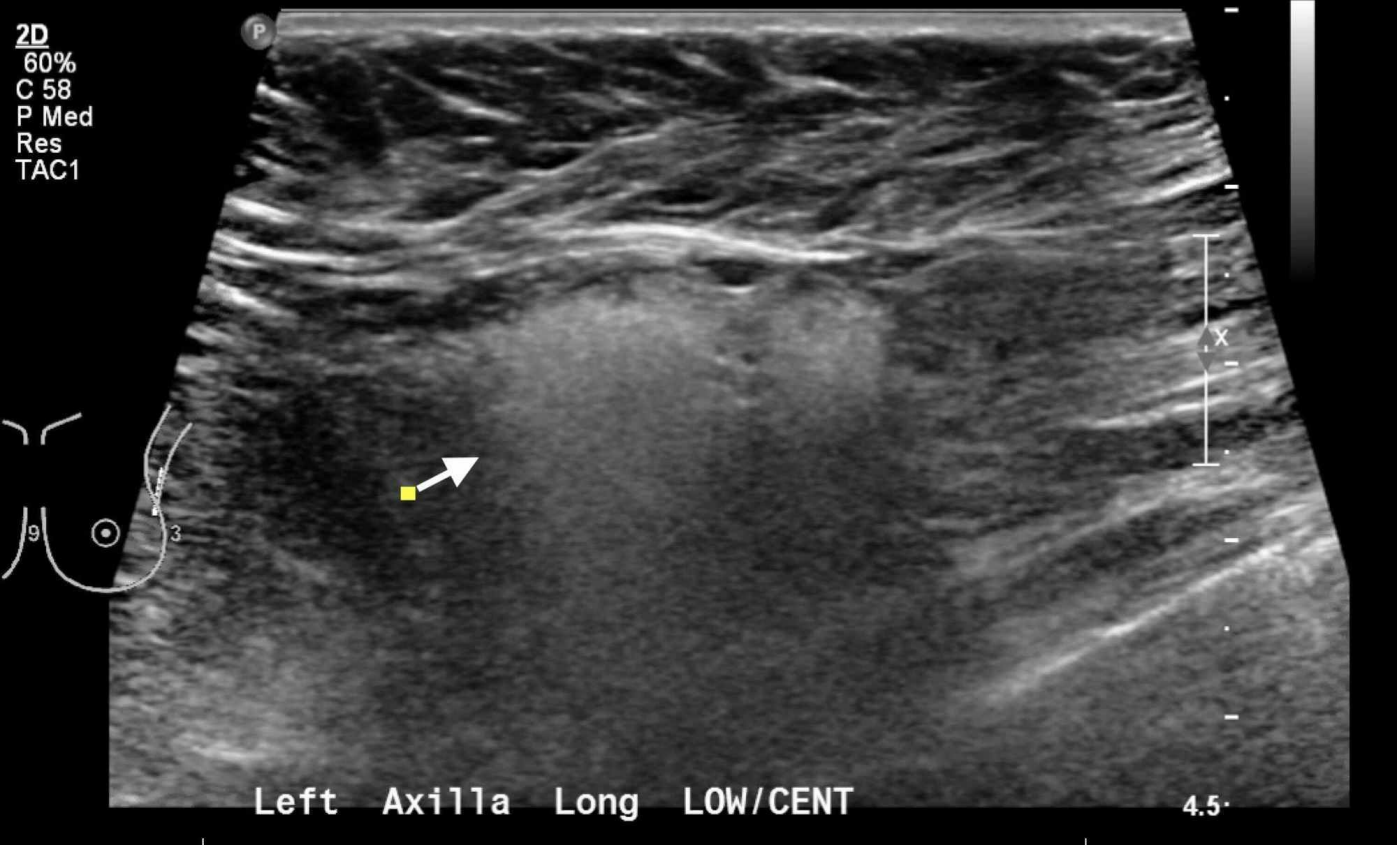
Preoperative ultrasound 2D ultrasound showing two left axillary lymph nodes with "sandstorm" appearance (white arrow) consistent with silicone lymphadenopathy

**Figure 2:**
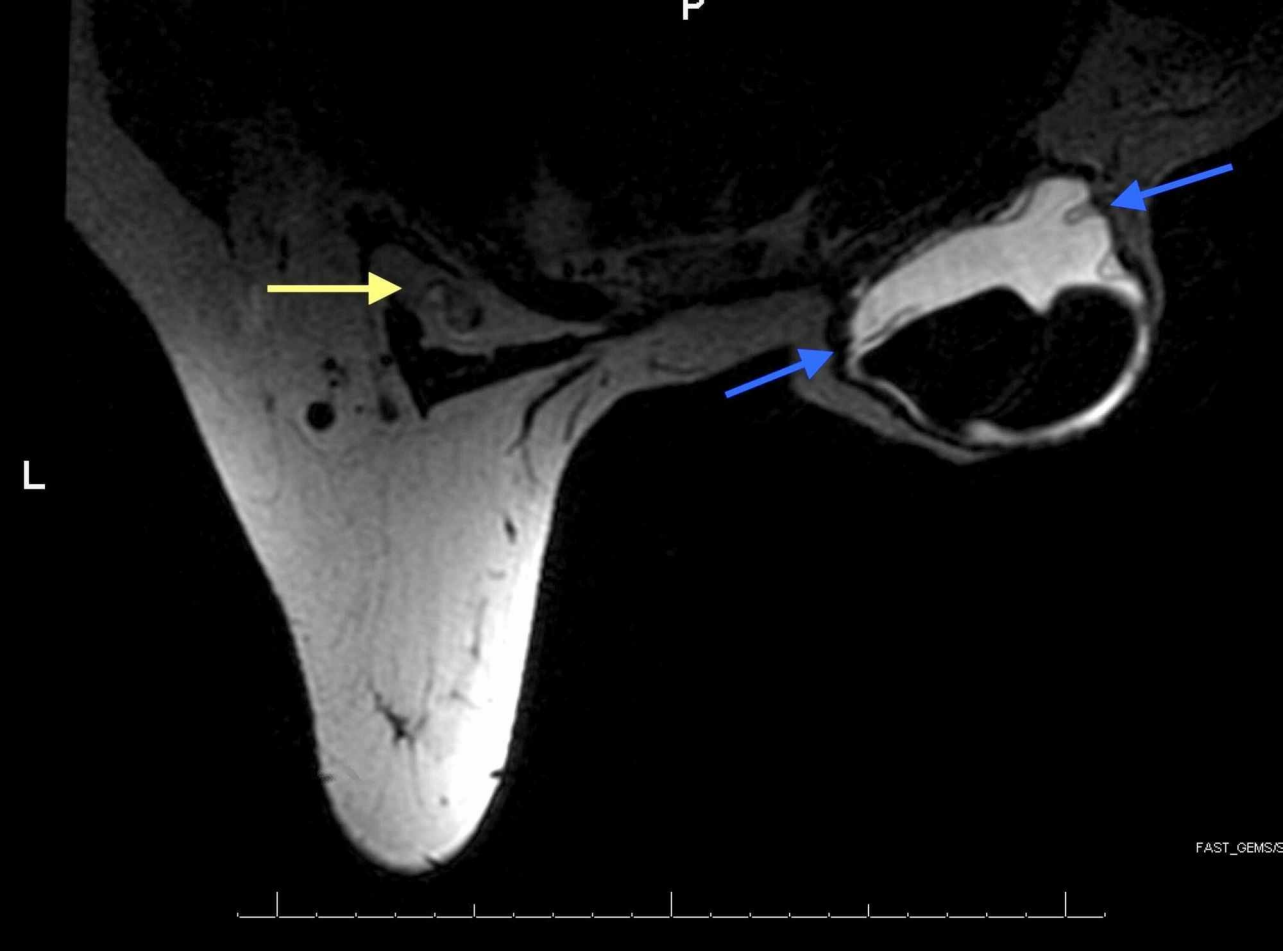
Preoperative MRI MRI shows multiple "keyhole signs" (blue arrows) within the silicone component of the Becker implant; silicone is seen in the contralateral interpectoral lymph node (yellow arrow) MRI: magnetic resonance imaging

Due to the abnormal mammogram findings, an ultrasound of the left breast and axilla was performed. It showed 1.3 x 1.0 x 1.0-cm and 1.0 x 0.9 x 0.9-cm central axillary lymph nodes with a "sandstorm" appearance, suggestive of silicone lymphadenopathy (Figure 1). An MRI was then performed with findings suggestive of right implant intracapsular rupture and silicone within the left axillary lymph nodes as well as left interpectoral Rotter’s nodal basin. Specifically, MRI findings identified a right breast dual lumen subpectoral implant with multiple keyhole signs at the superior aspect of the implant, free silicone present within the capsule adjacent to the implant consistent with intracapsular rupture, left interpectoral lymph node that appeared to contain silicone, and a 1.8-cm left axillary lymph node containing silicone corresponding to ultrasound findings (Figure 2).

Given the results of the radiologic findings without any abnormal physical exam findings, the patient elected to undergo removal of the right ruptured implant with capsulectomy and implant replacement. She elected not to have the left axillary lymph nodes removed and chose to continue surveillance of the axillary lymphadenopathy with no change noted on the one and two-year follow-up imagings. Intraoperative findings were consistent with MRI imaging showing rupture of the silicone component of the implant and loose silicone noted within the capsule. The silicone was removed, the pocket irrigated, capsule removed, and a 485-cc breast implant was placed (Sientra HSC Textured Round Moderate Plus Projection; Sientra, Santa Barbara, CA)

## Discussion

Clinical findings associated with implant rupture that would lead to evaluation include a change in breast shape, size or firmness, capsular contracture, palpable lumps, or breast pain [8]. Most implant ruptures are silent or do not manifest with clinically significant signs. Radiologic modalities such as ultrasound, mammography, CT, or MRI are used to assist in diagnosis. MRI is accepted as the modality of choice for evaluation of implant integrity with a sensitivity and specificity of over 90% for the detection of rupture [9]. The United States Food and Drug Administration recommends that patients with silicone gel implants undergo MRI screening three years postoperatively and at two-year intervals thereafter; however, compliance is low due to associated cost among other reasons. MRI provides the best visualization and identification of intracapsular implant disruption [13]. Yet, a meta-analysis has found improved accuracy of MRI for the detection of implant rupture in symptomatic patients versus asymptomatic patients [14]. As discussed earlier, rupture incidence increases after six to seven years, so it may be costly and less accurate to perform multiple MRIs to evaluate for rupture prior to six years. Classically, the most reliable MRI criterion for intracapsular rupture has been the linguine sign, which represents a collapsed implant shell floating within silicone gel. This sign is common for second-generation implants manufactured in the 1970s to late 1980s, but uncommon for third-generation implants due to their reinforced elastomer shell and more cohesive silicone gel [13]. Second-generation implants were characterized by a thin elastomer shell and softer gel filler to promote a more natural feel compared to firm first-generation implants [11]. A silicone droplet contained within radial folds, known as the keyhole, noose, or teardrop sign, is a common sign indicative of implant rupture in third-generation implants [13]. With regard to our case, MRI identified multiple keyhole signs along the superior aspect of the implant as well as loose silicone within the capsule in our patient, and the MRI findings were consistent with the operative findings.

The recommended selection of imaging modality depends on the reason or purpose for evaluation. If cancer of the breast is the reason for evaluation, it is recommended to have mammography performed with additional views (Eklund modified compression technique), which may be able to identify extracapsular rupture, but has low sensitivity for identification of intracapsular ruptures. If evaluation for implant rupture is of highest concern, MRI should be the modality of choice. However, ultrasound can be considered in a center where radiologists have sufficient experience to identify ruptures with confidence [13]. As part of her breast cancer surveillance, our patient had presented for routine screening mammography. No signs of implant rupture were noted on the right, and the left breast revealed benign findings with enlarged left axillary lymph nodes. Ultrasound was ordered to evaluate the enlarged lymph nodes, and a "sandstorm" appearance was noted within the lymph nodes, which is a recognized radiologic sign of silicone lymphadenopathy. This unexpected finding triggered evaluation for implant rupture with MRI. The MRI confirmed silicone within the left axillary lymph nodes and diagnosed a silent right breast intracapsular rupture.

The etiology of implant rupture is a multifactorial process with several contributing factors identified. Factors associated with implant rupture include surgical instrument damage (50-64%), unidentified opening/rent (no evidence of sharp instrument damage or shell fatigue wear), fold flaw, silicone swelling, and implant trauma (force to the chest or closed capsulectomy) [5]. While not a major cause of implant failure, fold failure has been further expanded upon by Brandon et al. by identifying different patterns of fold failure on microscopy leading to rupture in an effort to improve implant shell design [15].

The rate of rupture varies based on implant generation, implant age, manufacturer, and means of rupture identification. Currently, there are five generations of implants, and each generation has different gel properties and shell elastomer properties that have been modified with each generation by different manufacturers to improve the natural feel of the implant and reduce rupture risk as well as other complications. Devices of recent generations contain a more cohesive, highly crosslinked gel that is less likely to disseminate beyond the capsule compared to older generations. With regard to current-generation implants, prospective core clinical studies by breast implant manufacturers appear to have the strongest data on incidence [5]. Yet, there is no standardized method for statistical calculation or for reporting verified rupture rates without implant removal, which is an option patients may reject. There are currently three FDA-approved manufacturers in the United States for breast augmentation and reconstruction: Allergan, Mentor, and Sientra. The core studies included a cohort screened with MRI only and the other cohort evaluated by MRI if physical symptomology suggested rupture. Thus, the true rupture rate may be underestimated when screening by symptomology as most ruptures are often silent. Comparing MRI-only cohorts, Allergan Naturelle round and shaped 10-year rupture rate showed 35.4% and 12.4%, respectively, for the reconstruction group and 13% overall; Sientra nine-year rupture rate was 8.2% for primary reconstruction, and Mentor memory gel six-year rupture incidence was 3.8% for primary reconstruction group [16]. Our study included a patient with a Mentor Becker round Implant, which is a dual lumen implant composed of cohesive silicone gel in the outer chamber and saline in the inner chamber. Based on adjunct studies performed by Mentor, through five years, 7.5% of primary reconstruction patients and 10.4% of revision reconstruction patients experienced a rupture [17].

While a majority of implant ruptures are intracapsular, extracapsular free silicone migration has been the subject of many studies. Based on the Danish comprehensive national database of women studies using MRI over a two-year period, the majority of implant ruptures were intracapsular, and only 10% of intracapsular ruptures progressed to extracapsular ruptures, with 84% of extracapsular ruptures remaining stable over the two-year period [18]. Silicone migration beyond the breast capsule into breast parenchyma, chest wall musculature, abdominal wall, groin, back, pleural space, upper extremity, and axillary lymph nodes have been described with various associated symptoms [10,11]. It is well established with multiple studies that free silicone gel outside of the elastomer shell does not increase the risk of immunologic disorders [18].

Silicone lymphadenopathy is an uncommon finding of implant rupture that presents 6-10 years after implant placement based on current studies [11,19]. The actual incidence and prevalence are unknown with less than 180 cases noted in the literature. It does represent a normal physiologic response to foreign material. Migration of silicone into the surrounding breast tissue leads to a foreign-body reaction with silicone particles phagocytosed by multinucleated giant cells, leading to eventual scarring, granulomata formation, and eventual drainage via lymphatic system [19]. Silicone lymphadenopathy may present silently with radiologic signs only or clinically as a palpable mass in the axilla with or without signs of implant rupture, or it may mimic malignant lymphadenopathy [12]. The diagnosis of silicone lymphadenopathy can be established by radiology, core needle biopsy, fine needle aspiration, or open biopsy. Similar to the treatment of implant rupture, the removal of lymph nodes containing silicone is not considered necessary as it poses no significant health burden [8]. However, removal should be recommended for symptomatic patients and if clinically concerned for malignant lymphadenopathy [12]. Our case has highlighted that in a patient with a history of breast cancer, the differential diagnosis for regional lymph node involvement should include silicone lymphadenopathy as well as metastatic breast cancer.

A unique feature of this case is the identification of isolated axillary silicone lymphadenopathy contralateral to the ruptured reconstructed breast, which is the result of disrupted lymphatic drainage due to previous lymphatic dissection. Classically, the majority of lymphatics from the breast drain to the ipsilateral axillary nodes with minor drainage to the internal mammary, infraclavicular, supraclavicular, and interpectoral nodes at different ratios depending on the region of the breast. Following axillary lymph node dissection, alternate lymphatic pathways have been described, including internal mammary and contralateral lymph nodes [20]. Sato et al. compared patients with previous sentinel lymph node biopsy or axillary lymph node dissection with radiation and those without radiation, and found that aberrant drainage was significantly more common in patients with previous radiation. A high percentage of the aberrant drainage was observed to the contralateral axilla in the post-radiation group compared to patients without radiation [20]. Our patient had a history of previous radiation as well as axillary lymph node dissection, predisposing her to aberrant axilla lymph node drainage to the contralateral axilla and explaining the finding of silicone lymphadenopathy contralateral to her reconstructed breast.

## Conclusions

Axillary lymphadenopathy incidentally found on routine breast cancer surveillance can lead to significant patient angst. It is important to recognize free silicone migration in the differential diagnosis for regional lymphadenopathy in a post-mastectomy patient with implant-based reconstruction. As described in this unique case, post-mastectomy patients with previous axillary lymph node dissection and radiation may present with contralateral silicone lymphadenopathy. Our patient recognized that silicone lymphadenopathy is a benign finding and elected to undergo radiologic as well as clinical surveillance with no changes noted at the 24-month follow-up.
